# Time-Resolved Vibrational Analysis of Excited State Ab Initio Molecular Dynamics to Understand Photorelaxation: The Case of the Pyranine Photoacid in Aqueous Solution

**DOI:** 10.1021/acs.jctc.0c00810

**Published:** 2020-09-21

**Authors:** Maria Gabriella Chiariello, Greta Donati, Nadia Rega

**Affiliations:** † Dipartimento di Scienze Chimiche, Università di Napoli Federico II, Complesso Universitario di M. S. Angelo, via Cintia, I-80126 Napoli, Italy; ‡ CRIB Center for Advanced Biomaterials for Healthcare, Largo Barsanti e Matteucci, I-80125 Napoli, Italy

## Abstract

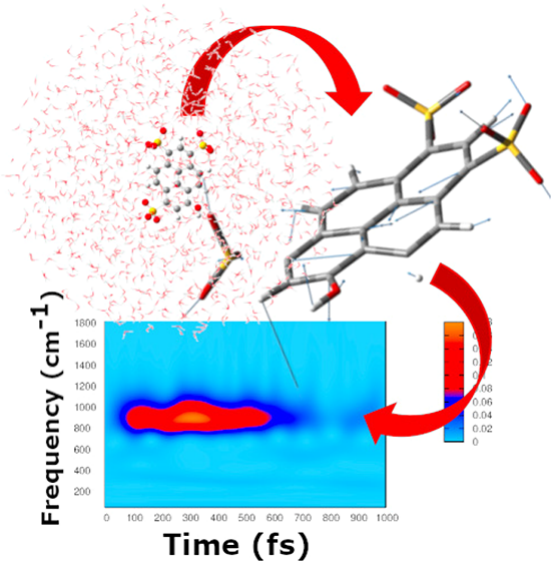

We present a novel time-resolved vibrational analysis for studying photoinduced nuclear relaxation. Generalized modes velocities are defined from ab initio molecular dynamics and wavelet transformed, providing the time localization of vibrational signals in the electronic excited state. The photoexcited pyranine in aqueous solution is presented as a case study. The transient and sequential activation of the simulated vibrational signals is in good agreement with vibrational dynamics obtained from femtosecond stimulated Raman spectroscopy data.

Nowadays, the employment of the high resolution time-resolved spectroscopic techniques makes possible the investigation of photoinduced chemical reactions on the time scale of nuclear motions. Specifically, vibrational spectroscopies are suitable to *watch* nuclear motions of molecules in real time upon excitation.^1−3^ Femtosecond stimulated Raman spectroscopy (FSRS), for example, is one of the promising experimental techniques capable of revealing at the atomistic level the reaction mechanism triggered by photoexcitation and, potentially, to unveil the nuclear-electronic coupling occurring with the electronic density redistribution.^4−6^

The resulting experimental spectra are often extremely complex, and the disentanglement of the information hidden in the signals is not a simple task. In this context, the vibrational dynamics provided by theoretical-computational approaches can be an excellent support to shed light on the nature of the vibrational modes giving rise to the observed experimental signals. In spite of the active research in the field,^7,8^ an integrated and well-established computational procedure able to provide a molecular interpretation of the vibrational photorelaxation phenomena is still unavailable.

The solution of the vibrational problem based on standard Hessian-based quantum mechanical approaches requires the localization of an energy minimum on the potential energy surface and becomes prohibitive to apply for a large system such as molecules in the condensed phase.^9,10^ An appealing alternative is represented by the generalized vibrational modes defined from ab initio molecular dynamics^11,12^ (AIMD) by using the covariance matrix of the Cartesian atomic velocities.^13,14^ This approach allows one to extract vibrational motions underlying the dynamics of molecules modeled in their realistic environment. Therefore, explicit solvent models can be adopted.^15−17^

The assumption in this case is that at any temperature 3N generalized molecular modes **Q** can be defined in such a way to correspond to uncorrelated linear momenta; namely they can be obtained by diagonalizing the **K** matrix of the mass weighted atomic velocities **q̇** with elements
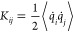
1where *i* and *j* run over the 3N atomic coordinates, and ⟨...⟩ indicates the average over the time.^18−21^

Composition of the generalized modes are given by the **K** eigenvectors collected in the unitary transformation matrix **L**. Projection of mass weighted atomic velocities along the modes gives us the time-resolved mode velocity vector **Q̇**(*t*), and vibrational frequency values can be obtained by Fourier transforming the corresponding autocorrelation functions.

The definition of generalized modes **Q**, unlike that of normal and quasi-normal ones,^22,23^ does not require a quadratic form of the potential, hence these collective coordinates correspond to molecular motions intrinsically anharmonic, showing anharmonic frequencies and coupling to other vibrations.^24^ This methodology has been successfully adopted for the vibrational analysis of molecular systems at the equilibrium, which can be confronted to steady-state vibrational spectra.^19,20,25^

In this work, we aim at extending the procedure above to the analysis of far from equilibrium processes, specifically the transient vibrational signals activated in relaxation processes at the electronic excited state (ES). Once thermodynamic equilibrium of a molecular system in the electronic ground state has been characterized by AIMD simulations and generalized mode analysis, a photorelaxation process can be simulated by a proper number of ES AIMD trajectories starting from suitable points (configurations and momenta) that represent the ground state equilibrium. During the relaxation, the time evolution of generalized modes **Q**_ES_ in the excited state can be obtained from mass weighted atomic velocities **q̇**_ES_ extracted and averaged from ES trajectories, according to the transformation
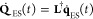
2Here we assume that the modes composition obtained in the ground state (given by **L**^†^) still hold in the excited state, as long as the relaxation has not led to a new arrangement of forces among nuclei and, as a consequence, to a new normal modes composition. This approximation is reasonably true in the ultrafast part of the relaxation and in proximity of the Franck–Condon region. The knowledge of relaxation times from experimental time-resolved spectra can also assist and validate the choice of this approach.

In order to obtain the vibrational frequency values along the time, we adopted a multiresolution vibrational analysis based on the Wavelet Transform (WT).^26−31^ WT has already been employed in combination with AIMD simulations, to disentangle the evolution of a simulated Stokes shift,^32^ of the dipole moment in exciton dynamics,^33^ and to analyze the phototriggered proton shuttle of green fluorescent protein in the time-frequency domain.^34^ In the present work, for the first time, we use WT to obtain transient vibrational signals corresponding to the **Q̇**_ES_(*t*) modes extracted from AIMD.

We adopt the continuous WT expression
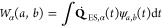
3where α runs over the 3N generalized modes.^35^ In this way, time dependent signals **Q̇**_ES_(*t*) are analyzed and decomposed in terms of wavelet basis ψ_*a*,*b*_. These are obtained from a so-called mother wavelet by dilatation and translation.
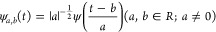
4

We chose the Morlet function as the mother wavelet. The scale parameter *a*, proportional to the inverse of frequency, regulates the dilatation and contraction of the mother wavelet and extracts the different frequencies hidden in the time-dependent signal. On the other hand, the translation of the wavelet basis, ruled by the *b* parameter, ensures the localization of the frequencies in the time domain. We plot the magnitude square of the transform |*W*_α_(*v*, *t*)|^2^ as the intensity of the instantaneous frequency contribution to the signal. As final result, we obtain power spectra of the generalized modes velocity **Q̇**_ES_, by retaining localization of each signal in both time and frequency domains. This approach allows one to monitor characteristic photoinduced vibrational dynamics in excited molecules.

The phototriggered vibrational dynamics of the 8-hydroxypyrene-1,3,6-trisulfonic acid (HPTS or pyranine, see Figure 1a) in water solution has been chosen as the pilot application of the method. Pyranine is a popular photoacid^36−39^ with the p*K*_a_ value lowered by 7 units upon electronic excitation. The excited state proton transfer (ESPT) reaction^40−45^ between the photoacid molecule, acting as the proton donor, and a nearby solvent water molecule occurs with time constants of 3 and 90 ps.^46^ Off-resonance FSRS experiments revealed that in the electronic excited state HPTS undergoes a transient (subpicosecond time scale) and sequential activation and decay of low frequency (<1200 cm^–1^) skeleton modes.^47,48^ This peculiar Raman activity precedes and possibly prepares the ESPT reactive event.

**Figure 1:**
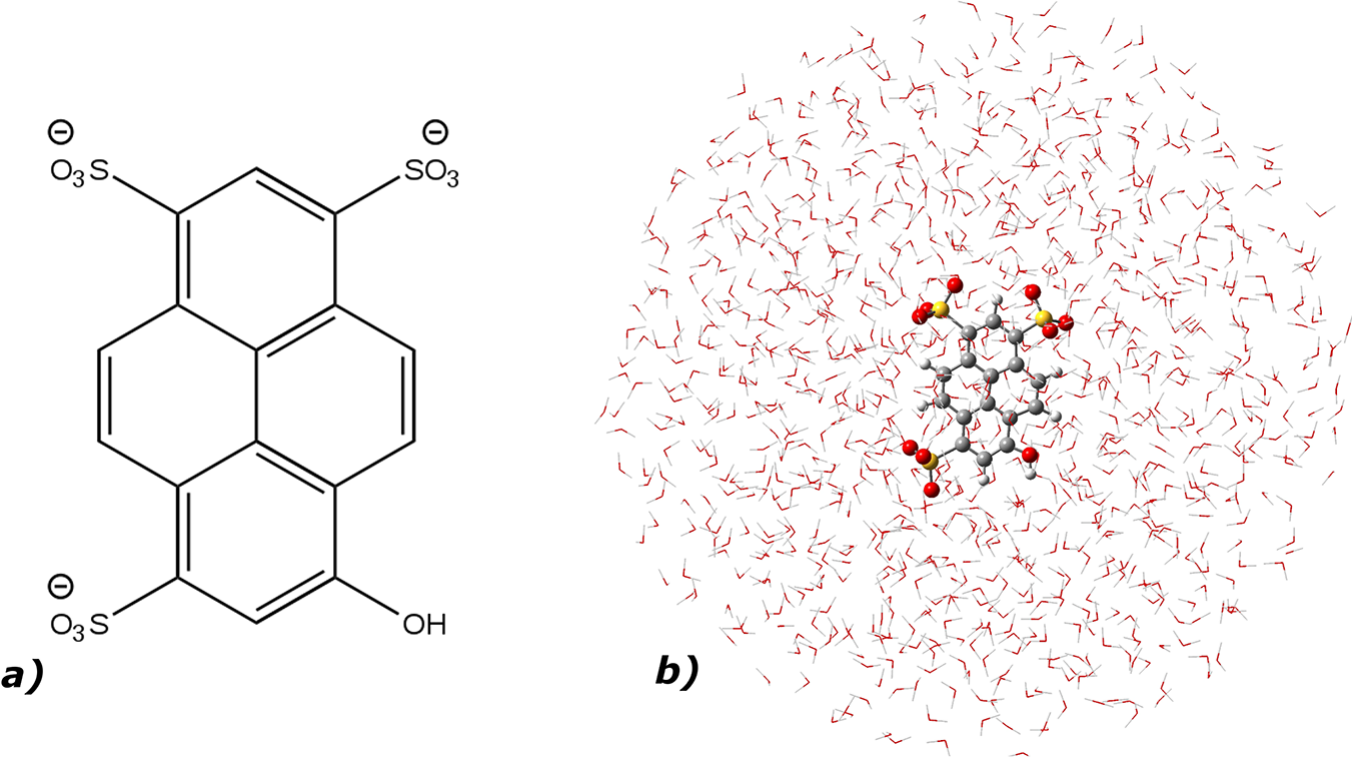
a) 8-Hydroxypyrene-1,3,6-trisulfonic acid (HPTS or pyranine) and b) HPTS in aqueous solution: in the hybrid implicit/explicit solvation model, HPTS is treated at the quantum mechanical level (DFT and TD-DFT level of theory for the ground and excited states, respectively), while the remaining explicit solvent molecules are modeled by molecular mechanics.

We adopted our method to analyze the transient vibrational relaxation of the photoexcited HPTS in a time window of 1 ps after the excitation. When experimental Raman activity over time is mainly ruled by the vibrational relaxation, vibrational dynamics simulated according to eqs 1–3 can retrace timing and patterns of Raman signals.

As depicted in Figure 1b, the pyranine has been placed at the center of a sphere filled by water molecules explicitly described with the TIP3P model in a flexible version,^49^ while a structureless solvent layer surrounds the explicit system.

By this hybrid explicit/implicit solvation method, which exploits nonperiodic boundary conditions,^15−17^ we could retain specific interactions between pyranine and the solvent and accurately reproduce the solvent dynamics in proximity of the solute.

In this model the pyranine was represented through Density Functional Theory^50−52^ (DFT) and Time Dependent (TD)-DFT^53−56^ to run AIMD trajectories in the electronic ground (S_0_) and excited (S_1_) states, respectively.^57,58^ In particular, five points (coordinates and momenta) were extracted from the S_0_ trajectory as starting configurations of just as many ES simulations.^59^ These trajectories were then used to perfom time-resolved vibrational analysis according to eqs 1–3. As support, quantum mechanical Hessian-based harmonic frequency calculations on S_0_ and S_1_ pyranine minimum energy structures in implicit aqueous solvent^60−62^ were also performed. All the calculations were carried out with the Gaussian16 suite program.^63^ Computational details are further given as Supporting Information (SI).

In the following, we discuss vibrational signals testifying the photorelaxation of the pyranine in aqueous solution after a π–π* excitation to the first singlet S_1_ state. We analyzed, in particular, those vibrational bands that show a complex dynamics according to off-resonance FSRS data, with signals appearing and disappearing in a tangled temporal sequence in the first hundreds of femtoseconds, i.e., the time necessary to complete the first important pyranine structural rearrangement.^47,64^

Table 1 summarizes the main features of the vibrational bands discussed in this work, namely the nature of the corresponding mode, the experimental frequency, and the experimental behavior over time (rise and decay time, oscillatory or monotone pattern).

**Table 1:** Mode Description, Experimental Frequency (cm^–1^), and Kinetics (fs) of Pyranine Vibrational Bands Analyzed in This Work^47^

mode	exp. freq	exp. rise time	exp. decay time	oscillating intensity
ring deformation + H out-of-plane	952	140	600	no
out-of-plane ring deformation	630	300	>1000	yes
ring deformation + COH rocking	362	650	>1000	no
vertical breathing	191	320	540	no

All of the vibrational modes have a collective nature, involving the motion of the whole four ring aromatic system. We considered the ring deformation modes at about 950, 630, and 360 cm^–1^ and the skeletal breathing at about 190 cm^–1^.

Figures 2 and 3 summarize analysis of these modes performed according to eqs 1–3, with **q̇**_ES_ obtained from excited state trajectories.

**Figure 2:**
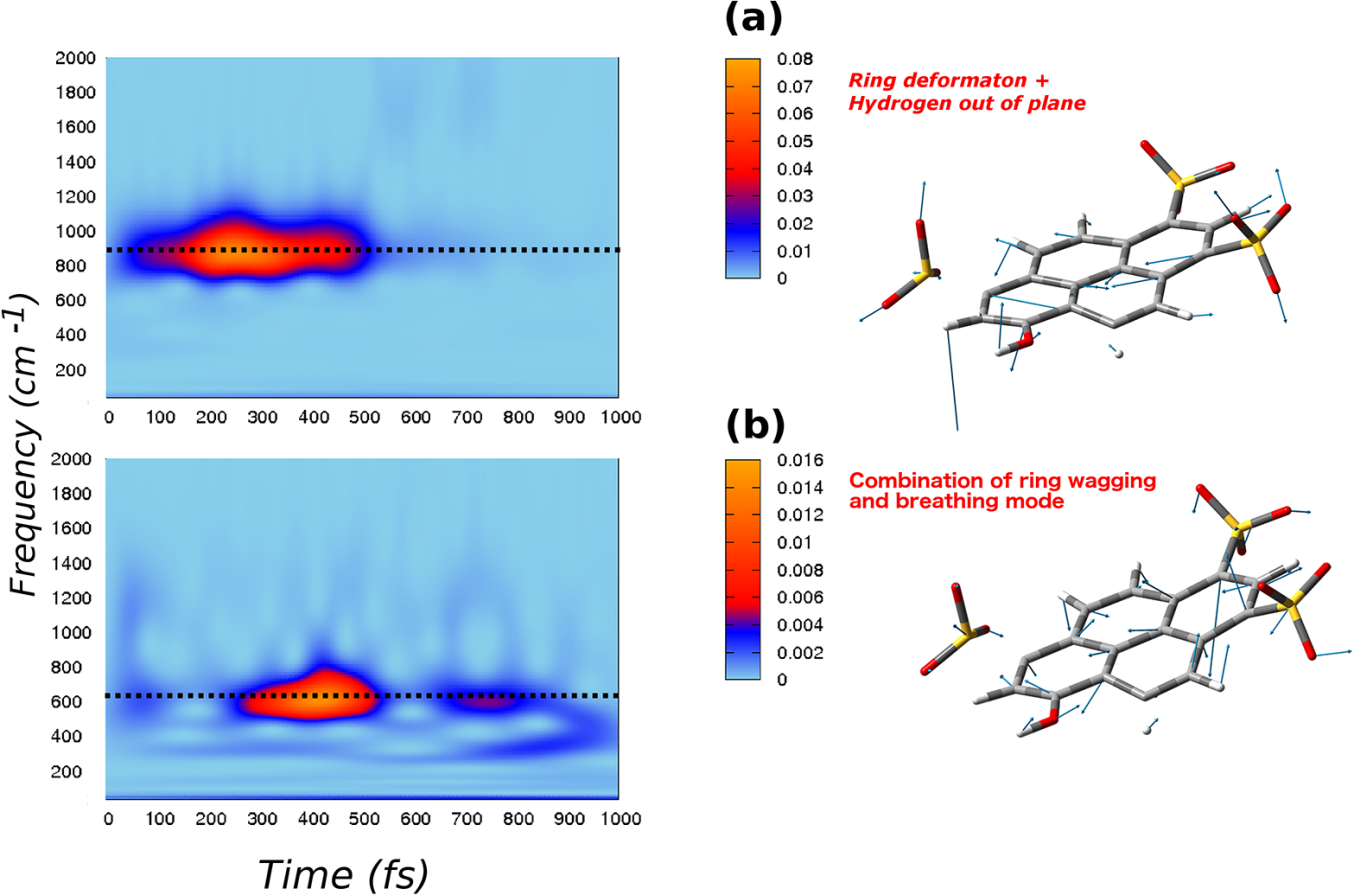
*Q* modes (right panels) and corresponding 2D wavelet power spectra (left panels). The color scale states for the intensity are in arbitrary units: a) a ring deformation mode combined with hydrogens out-of-plane motion, with an AIMD frequency at about 930 cm^–1^, an exp. value of 952 cm^–1^, an exp. rise time of 140 fs, and a decay time of 600 fs (from ref (47)) and b) a combination of ring wagging and breathing mode, with an AIMD frequency of 620 cm^–1^, an exp. value of 630 cm^–1^, and an exp. rise time of 300 fs.

**Figure 3:**
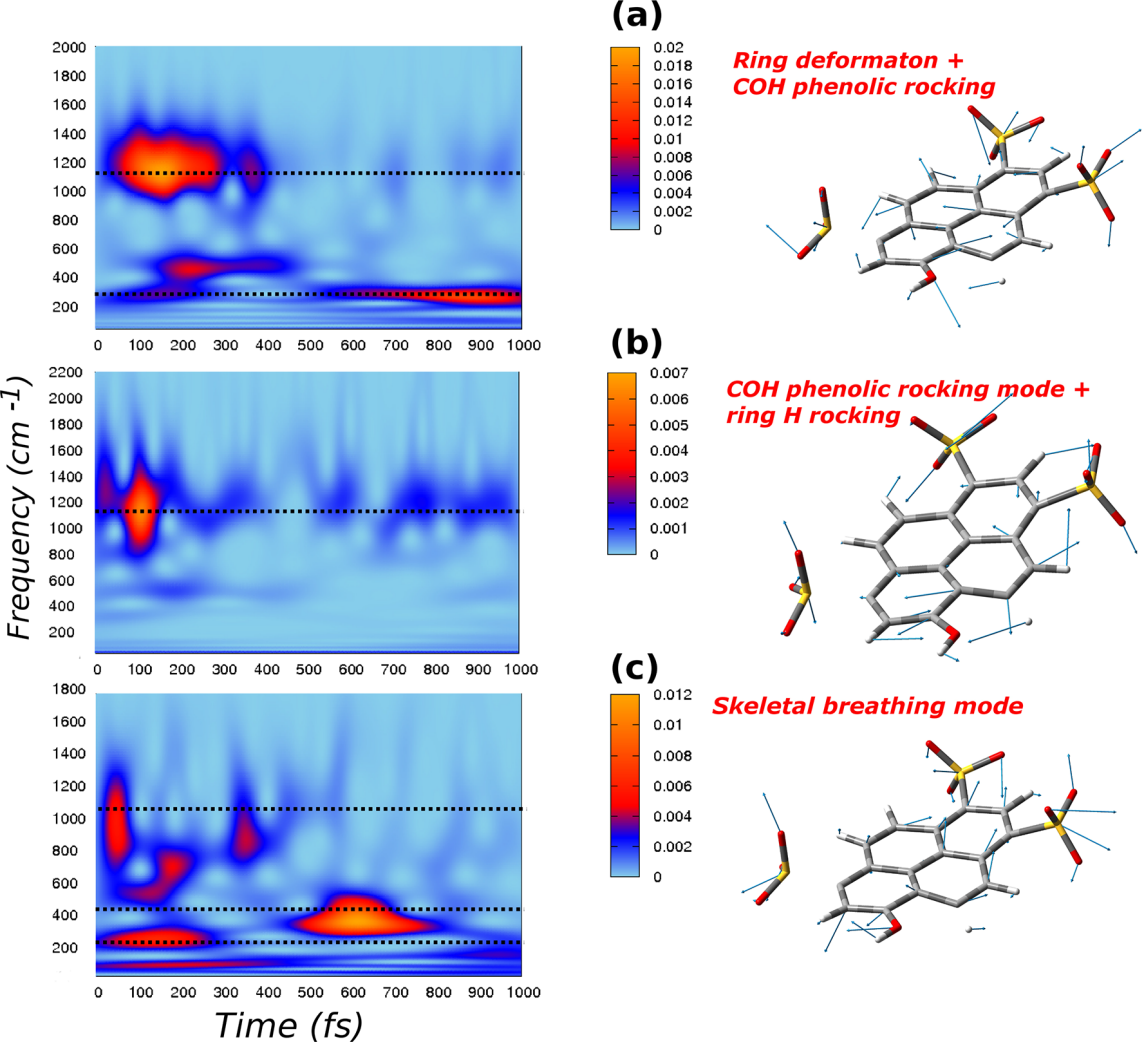
*Q* modes (right panels) and corresponding 2D wavelet power spectra (left panels). The color scale states for the intensity are in arbitrary units: a) ring deformation modes associated with a strong −COH phenolic rocking, with an AIMD frequency of 320 cm^–1^, an exp. value of 321 cm^–1^, and an exp. rise time of 680 fs; b) COH phenolic rocking combined with the rocking motion of the nearby ring hydrogen, with an AIMD frequency of 1156 cm^–1^ and an exp. value of 1154 cm^–1^; and c) a skeletal breathing mode, with an AIMD frequency of 190 cm^–1^, an exp. value of 191 cm^–1^, and an exp. decay time of 540 fs.

Generalized mode composition obtained according to the **L**^†^ transformation is shown in the right panels of Figures 2 and 3, while wavelet spectra of corresponding **Q̇**_ES_ velocities are reported in the left panels as 2D maps. Spectra are plotted in the frequency range of 0–2000 cm^–1^, because at higher frequencies there are no signals of considerable intensity.

The generalized mode in the right panel of Figure 2a is an in plane ring deformation with an important hydrogen out-of-plane component. It corresponds to the normal mode with harmonic frequency calculated in S_1_ of 970 cm^–1^ (see Figure S5 in the SI) and to the vibrational band observed at about 950 cm^–1^ by FSRS of Table 1. By inspection of the wavelet spectrum of this mode in the left panel of Figure 2a, a well isolated band centered above 930 cm^–1^ starts to rise after the electronic excitation, at about 100 fs. Later, the signal shows a decay at the time of 600 fs. This behavior shows a very good agreement with the experimental evidence of kinetics constants of 140 fs (rise) and 600 fs (decay) of the deformation band as reported in Table 1.

In the right panel of Figure 2b is instead depicted the composition of the generalized mode given by the combination of ring wagging and breathing modes with both in plane and out-of-plane ring deformations. This mode matches the normal mode in Figure S4 with harmonic S_1_ frequency at 660 cm^–1^, while the corresponding experimental FSRS band is recorded at about 630 cm^–1^ (see Table 1).

By confronting the 2D wavelet map in the left panel of Figure 2b with data in Table 1, the experimental frequency and rise time of 300 fs are well reproduced for this vibrational band. This mode has a decay time longer than 1 ps, and it is characterized by an oscillating intensity behavior over the time. Indeed, the wavelet spectrum shows that at the time of about 600 fs the signal starts to decrease, and then it raises again.

Spectra of generalized modes obtained by the present procedure can show signals at different frequencies due to the intrinsic anharmonicity. As a matter of fact, anharmonicity has been proven to be responsible for the coupling between high and low frequency modes in time-resolved vibrational signals.^24,65^ In particular, low frequency vibrations at 360 and 190 cm^–1^, with time-resolved vibrational analysis reported in Figure 3, show quite complex and informative spectra.

The signal at 360 cm^–1^ (see Figure 3a) is associated with the deformation mode experimentally found at the frequency of 362 cm^–1^, and it appears at 600 fs. We note again the very good agreement with the experimental frequency, as well as the rise time of 650 fs. In the same spectrum, another important signal is centered around 1156 cm^–1^. That is basically the −COH phenolic rocking mode, experimentally found at 1154 cm^–1^. The 360 cm^–1^ mode is overall composed of a four ring collective deformation and a strong phenolic −COH motion. Nevertheless, the −COH rocking mode alone is located at 1156 cm^–1^, and it was isolated and shown with the wavelet map in Figure 3b. During the dynamics simulation, the sampling of the 360 cm^–1^ mode involves also the −COH rocking motion, and, as a consequence, the wavelet spectrum shows a contribution from the band at 1156 cm^–1^. A further contribution at about 500 cm^–1^, not experimentally observed, is simulated in the spectrum of Figure 3a.

Lastly, in Figure 3c, the composition and wavelet spectrum of the lower frequency ring breathing mode is shown. A component below 200 cm^–1^, associated with the breathing mode, is easily recognizable. Following the electronic excitation, this mode quickly starts to rise showing a very short lifetime, with a decay at about 300 fs. In addition, the spectrum shows another important band appearing at about 500 fs in the 390–500 cm^–1^ region. This latter contribution can be associated with a mode experimentally found at 460 cm^–1^, with a rise time of about 600 fs. From the static frequencies calculation, the mode at 456 cm^–1^ seems to be very similar to the breathing mode in terms of collective motion of the four aromatic ring systems (see Figure S3). The AIMD simulation made possible the sampling and isolation of the 191 cm^–1^ breathing mode, that is naturally and sequentially coupled to the 460 cm^–1^. In the excited state wavelet spectrum, we can observe the band at 460 cm^–1^ appearing simultaneously with the breathing decay, qualitatively reproducing the experimental rise time of 600 fs. This finding has to be compared with the experimental assignment of a signal at 460 cm^–1^ to the deprotonated HPTS chromophore. The excited state trajectories^64^ show indeed a shorter bond between the phenolic group of pyranine and hydrogen bonded water molecule (i.e., the proton donor–acceptor pair). The 460 cm^–1^ transient mode seems to be rather characteristic of the photoexcited HPTS protonated chromophore. A further analysis of the composition of the breathing mode shows an important contribution localized on the phenolic group, i.e., the COH phenolic rocking motion. The wavelet map in Figure 3c shows indeed also a signal localized at 1156 cm^–1^, as already observed in the spectrum of 360 cm^–1^ mode. Hence, the three components of vertical breathing (190 cm^–1^), horizontal breathing (460 cm^–1^), and COH rocking (1156 cm^–1^) have been sampled together.

In summary, we propose a new computational strategy for the investigation of ultrafast nuclear photodynamics. The present method is able to provide an accurate picture of the time evolution of the photoactivated vibrational modes, matching in many cases the kinetics time constants of the experimental signals. As the first successful application, we considered the case of pyranine photoacid, where nuclear relaxation is finely controlled by a sequential and characteristic activation of low frequency modes (<1000 cm^–1^). This complex vibrational activity, observed by the FSRS experiments, is mostly reproduced in our simulations.

The method can be generalized and adopted for the study of photoinduced reactions. In particular, a promising extension of the approach would be the prediction of the anharmonic coupling between vibrational modes. The quantitative analysis of the oscillatory paths of time-resolved signals would reveal indeed the anharmonic coupling between frequencies.^24,65^

## Supporting Information Available

Computational details and frequency values and composition of vibrational modes computed at ground and first excited state energy minimum structures (PDF)
