# Supplementary material for: Time-Resolved Vibrational Analysis of Excited State Ab Initio Molecular Dynamics to Understand Photorelaxation: The Case of the Pyranine Photoacid in Aqueous Solution
Source: J Chem Theory Comput. 2020 Sep 21;16(10):6007–13. doi: 10.1021/acs.jctc.0c00810 (PMC8011922; doi:10.1021/acs.jctc.0c00810)
Supplement: Supplementary file 1 — ct0c00810_si_001.pdf [file ct0c00810_si_001.pdf]

Supporting Information: A Time-Resolved Vibrational Analysis of Excited State Ab-Initio Molecular Dynamics to Understand Photorelaxation: the Case of the Pyranine Photoacid in Aqueous Solution

Maria Gabriella Chiariello,[†] Greta Donati,[†] and Nadia Rega[*,†,‡]

†*Dipartimento di Scienze Chimiche, Università di Napoli Federico II, Complesso Universitario di M.S.Angelo, via Cintia, I-80126 Napoli, Italy*

‡*Italian Institute of Technology, IIT@CRIB Center for Advanced Biomaterials for Healthcare, Largo Barsanti e Matteucci, I-80125 Napoli, Italy*

E-mail: nadia.rega@unina.it

Contents

Computational details

Table S1: AIMD and harmonic frequencies computed at the ground and excited state minimum energy structure..

Figure S1: Composition of the breathing mode in the ground and excited state minimum.

Figure S2: Composition of the ring deformation + COH phenolic rocking mode in the ground and excited state minimum.

Figure S3: Composition of the horizontal breathing in the ground and excited state minimum.

Figure S4: Composition of the ring wagging and breathing combination mode in the ground and excited state minimum.

Figure S5: Composition of the ring deformation + H out of plane mode in the ground and excited state minimum.

Figure S6: Composition of the CHO phenolic rocking mode in the ground and excited state minimum.

Computational details

The pyranine solute was located at the center of a water molecules sphere of a radius 19 Å; the structureless solvent sourrounding the explicit sphere completes the hybrid explicit/implicit solvation model. Non periodic boudary conditions account for the interactions between the explicit molecular system and the implicit bulk solvent with both the electrostatic and dispersion-repulsion interactions. The solvent molecules are explicitely represented by the TIP3P water model, while the implicit bulk solvent by the polarizable continum model, in conductor-like version (C-PCM). The explicit system itself is treated at different levels of theory according to the hybrid QM/MM ONIOM extrapolation scheme. In particular, the pyranine molecule is described by DFT and TD-DFT in the ground (S_0) and S_1 excited state , respectively, by adopting the global hybrid B3LYP functional and the 6-31g(d,p) basis set. The so-built energy potential ruled the AIMD simulations in both ground and excited state. The ground state sampling was performed by means of the atom-centered density matrix propagation (ADMP) method. After 5 ps of equilibration, the trajectory was collected for 10 ps with a time step of 0.2 fs, keeping a constant temperature of 298 K. From the ground state trajectory, five configurations rappresentative of the equilibrium, with both coordinates and momenta have been chosen as starting points fot the propagation of the excited state dynamics. In order to collect the excited state trajectories, a Born-Oppenheimer ab-initio dynamics was instead employed, where the ecxited state energies and gradients were calculted on-the-fly by TD-DFT in its linear response formalism. All the ground and excited state AIMD trajectories, used for the wavelet-based vibrational analysis, were generated using the Gaussian16 suite of programs. The 6-31g(d,p)/b3lyp/CPCM level of theory was also adopted for the calculation of the harmonic vibrational frequencies of the pyranine molecule in the ground and excited state minimum energy structure. The effects of the solvent was included by means of the C-PCM model.

The wavelet transform (WT) is a multiresolution analysis, i.e. the analyzing time window is adapted like a *zoom lens* to the simulated signal. WT is capable of capturing both low and high frequencies contribution with an optimal resolution. The frequency resolution ($\Delta\nu$) is inversely proportional to the time resolution ($\Delta\tau$), according to the ($\Delta\nu\Delta\tau \geq 1/(4\pi)$).

We plotted the magnitude square of the transform $|W(v,t)|^2$. The intensities are reported in arbitrary units and the values of $|W(v,t)|^2$ represents the contribution to the signal of the instantaneous frequency. The intensity therefore is the results of the wavelet integration of the time dependent signal. Every time dependent vibrational signal ($\mathbf{\dot{Q}_{ES}(t)}$) (see Equation 2 and 3 of the main text) is wavelet transformed, therefore the signal intensities cannot be compared among different wavelet spectra.

Table S1: AIMD and harmonic frequencies in cm^{-1} of the principal vibrational modes. Harmonic frequencies refer to the S_0 and S_1 minimum energy structure computed at B3LYP/6-31g(d,p)/C-PCM level of theory.

	Exp	AIMD S_0	Harmonic S_0	Harmonic S_1
Vertical breathing	191	190	217	217
Ring deformation + COH rocking	362	360	349	349
Horizontal breathing	460		461	459
Out of plane ring deformation	630	620	660	662
Deformation + H out-of-plane	952	910	981	976
COH rocking + H ring rocking	1138/1154	1156	1156	1153

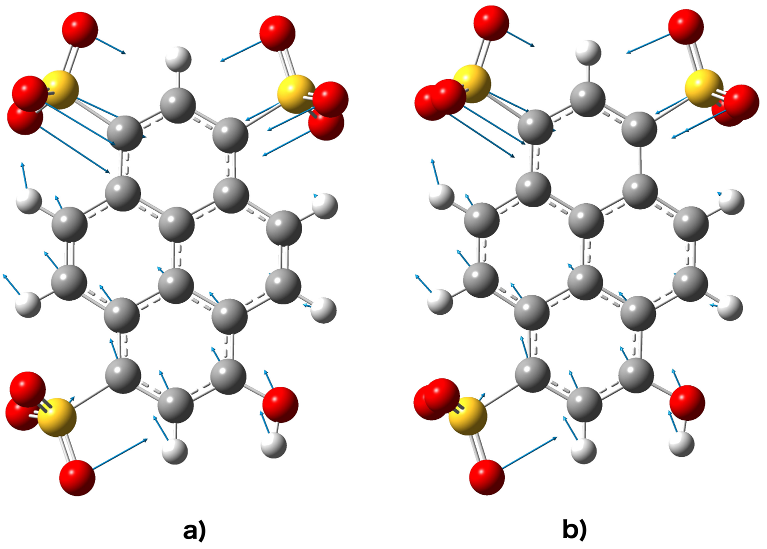

Figure S1: Composition of the skeletal breathing mode calculated in the a) S_0 (217 cm^{-1}) and b) S_1 (217 cm^{-1}) minimum.

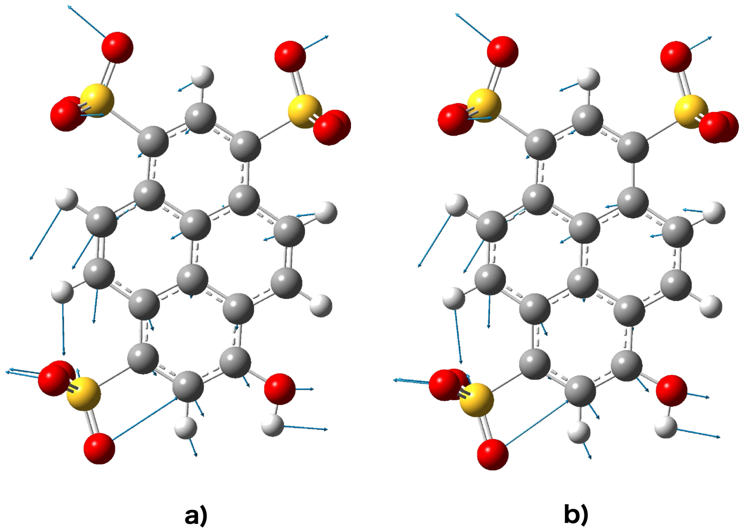

Figure S2: Composition of the ring deformation associated to COH phenolic rocking mode calculated in the a) S_0 (349 cm^{-1}) and b) S_1 (349 cm^{-1}) minimum.

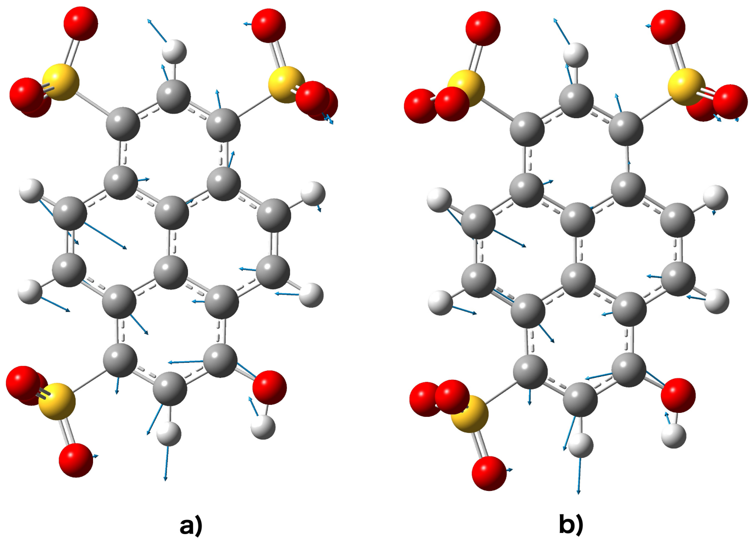

Figure S3: Composition of the horizontal breathing calculated in the a) S_0 (461 cm^{-1}) and b) S_1 (459 cm^{-1}) minimum.

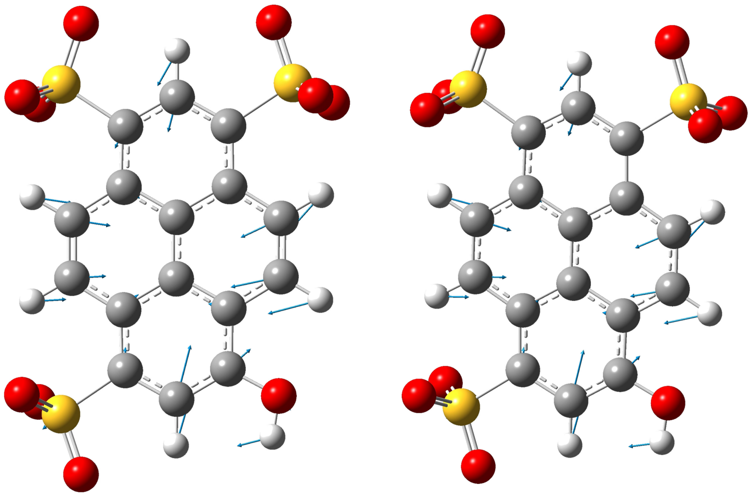

a) b)

Figure S4: Composition of the ring wagging and breathing combination mode calculated in the a) S_0 (660 cm^{-1}) and b) S_1 (662 cm^{-1}) minimum.

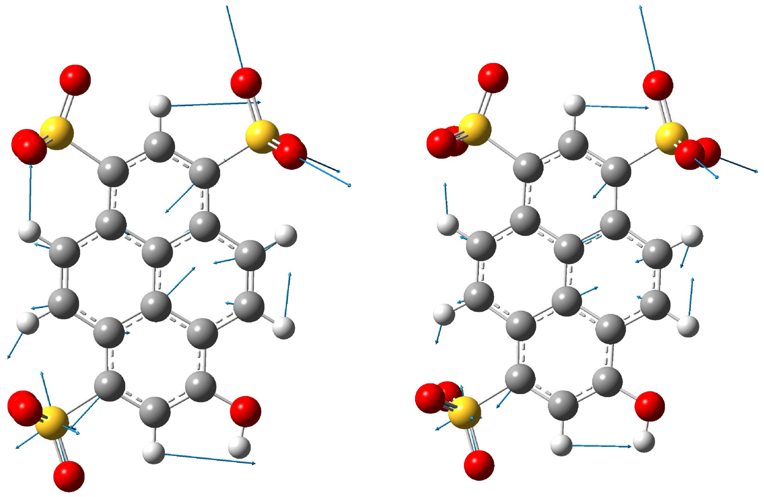

a) b)

Figure S5: Composition of the ring deformation associated to Hydrogen out of plane mode calculated in the a) S_0 (981 cm^{-1}) and b) S_1 (976 cm^{-1}) minimum.

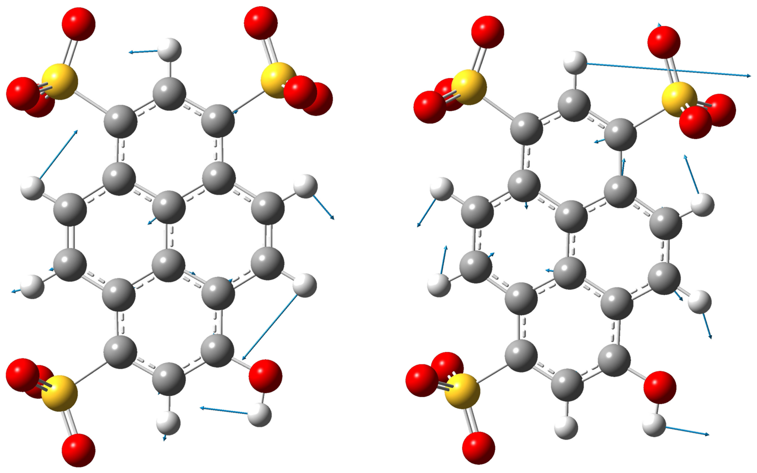

a) b)

Figure S6: Composition of the CHO phenolic rocking associated to nearby hydrogen rocking mode in the a) S_0 (1156 cm^{-1}) and b) S_1 (1153 cm^{-1}) minimum.
